# Membrane-Based CO_2_ Capture Across Industrial Sectors: Process Conditions, Case Studies, and Implementation Insights

**DOI:** 10.3390/membranes15070200

**Published:** 2025-07-02

**Authors:** Jin Woo Park, Soyeon Heo, Jeong-Gu Yeo, Sunghoon Lee, Jin-Kuk Kim, Jung Hyun Lee

**Affiliations:** 1 Energy Storage Research Department, Korea Institute of Energy Research, Daejeon 34129, Republic of Korea; bashal0907@kier.re.kr (J.W.P.);; 2 Chemical Engineering Department, Hanyang University, Seoul 04763, Republic of Korea; 3 Division of Energy Engineering, KIER School, University of Science and Technology, Daejeon 34129, Republic of Korea; 4 CCS Research Department, Korea Institute of Energy Research, Daejeon 34129, Republic of Korea

**Keywords:** membrane process, carbon dioxide capture, process demonstration

## Abstract

Membrane-based CO_2_ capture has emerged as a promising technology for industrial decarbonization, offering advantages in energy efficiency, modularity, and environmental performance. This review presents a comprehensive assessment of membrane processes applied across major emission-intensive sectors, including power generation, cement, steelmaking, and biogas upgrading. Drawing from pilot-scale demonstrations and simulation-based studies, we evaluate how flue gas characteristics, such as CO_2_ concentration, pressure, temperature, and impurity composition, govern membrane selection, process design, and operational feasibility. Case studies highlight the technical viability of membrane systems under a wide range of industrial conditions, from low-CO_2_ NGCC flue gas to high-pressure syngas and CO_2_-rich cement emissions. Despite these advances, this review discusses the key remaining challenges for the commercialization of membrane-based CO_2_ capture and includes perspectives on process design and techno-economic evaluation. The insights compiled in this review are intended to support the design of application-specific membrane systems and guide future efforts toward scalable and economically viable CO_2_ capture across industrial sectors.

## 1. Introduction

Global CO_2_ emissions, driven by human activities, have significantly accelerated climate change, raising atmospheric concentrations from 316 ppm in 1960 to a projected 426 ppm by 2025 and contributing to a 1.1 °C rise in average global temperature since pre-industrial times [1,2]. The rise in greenhouse gas emissions—primarily from fossil fuel use, deforestation, and agricultural expansion—has intensified climate-related impacts and highlighted the urgent need for technologies that can directly reduce or remove atmospheric CO_2_ [3,4,5,6,7,8,9,10].

Accordingly, CO_2_ capture technologies have been developed over the past decades, and efforts to effectively capture, store, and utilize CO_2_ emitted from various industrial sectors are still ongoing. For instance, the CO_2_ concentration in flue gas emitted from power plants, manufacturing facilities, and biogas upgrading processes shows a wide range, ranging from approximately 4% to 50% (Table 1) [11,12]. In addition, the presence of secondary components and impurities, together with the need for pretreatment, can significantly affect the efficiency of the capture process [13]. Accordingly, process design optimized for each condition is essential to achieve efficient CO_2_ capture across a range of technological options.

A range of CO_2_ capture technologies—including absorption, adsorption, membrane separation, cryogenic separation, and biological approaches—have been developed, each offering distinct advantages and limitations. As summarized in Table 2, while chemical absorption is effective for large-scale emitters, it incurs high solvent regeneration costs [1,14,15,16]. Adsorption requires substantial thermal input [17,18,19,20], cryogenic methods yield high-purity CO_2_ at high energy costs [21,22,23], and biological capture, though eco-friendly, is limited by slow uptake and large space requirements, underscoring the need for an integrated approach tailored to application-specific demands [24,25].

Membrane-based CO_2_ separation offers several advantages over conventional capture technologies, such as ease of operation, low energy consumption, and system simplicity [26,27,28,29,30,31]. Unlike absorption or adsorption methods, membranes enable CO_2_ separation without chemical reactions or solvent regeneration, resulting in a more environmentally benign and operationally straightforward process. Their compact design allows for easy integration into industrial systems, making them particularly suitable for decentralized or small-scale emission sources where conventional large-scale systems may not be feasible [32]. Additionally, membrane performance becomes more favorable when the CO_2_ concentration in flue gas exceeds 20%, as higher partial pressure enhances the driving force for selective gas permeation, leading to improved separation efficiency [12,33,34]. Therefore, the selection of an appropriate CO_2_ capture technology should be guided by the composition, temperature, and environmental characteristics of the target flue gas, as each method only demonstrates commercial viability when applied under conditions that maximize its advantages. This review focuses on industrial case studies of membrane-based CO_2_ capture processes, analyzing their configurations, performance metrics, and applicability across various emission sources. While both membrane gas absorption (e.g., membrane contactors) and membrane gas separation have been investigated for CO_2_ capture, this review focuses exclusively on the latter. Membrane gas absorption utilizes a liquid absorbent at the gas–liquid interface within porous membranes and is governed by reactive mass transfer mechanisms. In contrast, membrane gas separation relies on selective gas phase permeation through dense or composite membranes. Due to fundamental differences in transport mechanisms, operational configuration, and scalability, membrane absorption is excluded from the scope of this review, except for brief contextual comparisons where relevant.

## 2. Membrane Process for CO_2_ Capture

Membrane-based gas separation proceeds through three key steps: gas sorption at the feed side, diffusion across the membrane driven by a concentration gradient, and desorption at the permeate side. The process enables selective CO_2_ separation based on differences in gas permeabilities, which are governed by the physicochemical characteristics of the membrane material [35,36,37]. CO_2_ transport is driven by the pressure differential between the feed and permeate sides, and the flux can be modulated using compressors or vacuum pumps depending on the process’s configuration [38,39,40,41]. The overall performance of membrane systems is primarily influenced by the operating conditions, such as the feed pressure, permeate pressure, flow rate, and temperature [41,42,43,44,45,46,47,48,49,50].

Notably, trade-offs often arise between permeance and selectivity, as well as CO_2_ purity and recovery, necessitating precise optimization of membrane process parameters. Among the most influential factors is the choice of membrane material, which determines critical performance metrics, such as permeability, selectivity, and long-term stability. Membranes are fabricated in various structural formats, including thin-film composite (TFC) and facilitated transport membranes (FTMs), but they are broadly classified by their base materials into three categories, polymers, ceramics, and mixed-matrix membranes (MMMs), each tailored to specific operating environments.

Polymeric membranes, such as those made from polysulfone, polyimide, and Pebax, are commonly used due to their favorable CO_2_ selectivity and ease of processing. However, they are susceptible to plasticization and thermal aging degradation. Numerous studies have shown that strategies like polymer blending copolymerization can enhance mechanical and thermal stability [51]. Ceramic membranes, composed of materials like silica or alumina, offer exceptional resistance to heat and chemical corrosion, making them suitable for harsh conditions, such as those found in pre-combustion CO_2_ capture. Despite these advantages, their brittleness, water sensitivity, and high production costs have limited large-scale deployment [52,53]. MMMs that embed inorganic fillers, such as metal–organic framework (MOFs) or zeolites, into polymer matrices aim to combine the mechanical flexibility of polymers with the superior selectivity of inorganic materials. Recent advancements have demonstrated that well-designed MMMs can surpass the traditional Robeson upper bound, particularly in post-combustion capture scenarios [54]. This alignment is critical not only because of process compatibility but also due to intrinsic material limitations that impose performance boundaries [55].

While operational parameters are crucial for process optimization, intrinsic material limitations often impose more fundamental constraints on membrane performance. In particular, the inherent trade-off between permeability and selectivity remains a major challenge in the development of high-performance membranes. A well-recognized bottleneck in membrane-based CO_2_ separation is the permeability–selectivity trade-off, often visualized by the Robeson upper bound. This empirical relationship highlights that enhancing gas permeability typically leads to a loss in selectivity, making it difficult to achieve both high CO_2_ flux and high purity simultaneously. Such limitations are especially critical in industrial applications where low CO_2_ partial pressures, high flow rates, and economic constraints demand both performance and scalability. For instance, low driving forces necessitate membranes with high selectivity, but this often comes at the expense of permeance, requiring larger membrane areas and higher compression energy. Recent materials research, however, has shown promising approaches to mitigating this trade-off. N. Habib et al. demonstrated that incorporating ionic-modified metal–organic frameworks with 177 fillers into Pebax membranes significantly enhanced CO_2_/N_2_ selectivity, exceeding the Robeson upper bound under simulated flue gas conditions [56]. Similarly, a study reported ultrathin Polymethyl methacrylate composite membranes with improved H_2_/CO_2_ performance [57], while A. E. Amooghin et al. utilized MXene-based mixed-matrix membranes to achieve simultaneous improvements in both permeability and selectivity [58]. These findings emphasize that overcoming the permeability–selectivity trade-off is not only a matter of material innovation but also essential for ensuring the industrial feasibility of membrane-based CO_2_ capture systems.

Numerous studies have emphasized that the operating conditions of membrane systems significantly influence CO_2_ capture performance beyond the intrinsic properties of the membrane itself. For example, H. Wu et al. investigated the effects of the feed flow rate, pressure, CO_2_ concentration, and module configuration on system performance using spiral-wound FTMs composed of polypropylene, PET, and polyester nonwoven fabric [13]. Their findings showed that membrane area and energy demand varied substantially with feed gas composition and flow rate, even with identical membrane modules. Similarly, K. T. Woo et al. evaluated performance under different pressure ratios and CO_2_ concentrations, reporting that both purity and recovery improve with increased pressure ratio up to a limit, beyond which energy consumption becomes a constraint [59]. They also identified a complex trade-off between the feed flow rate and separation performance. Notably, they concluded that single-stage membrane systems cannot achieve high-purity CO_2_ recovery from typical post-combustion flue gases (10–20% CO_2_), underscoring the need for multi-stage configurations. H. Y. Hwang et al. further demonstrated that the optimization of temperature and feed pressure could double the permeance in a polysulfone membrane system, even without changing the membrane material [60]. Collectively, these findings highlight that membrane process efficiency is highly sensitive to operational variables and that rigorous process optimization is essential for maximizing both performance and energy efficiency.

Several simulation studies have been conducted to optimize membrane-based CO_2_ capture processes, focusing on parameters like the membrane area, energy consumption, and operating conditions. B. T. Low et al. evaluated the effects of membrane material properties and process variables under post-combustion conditions [61]. Their results indicated that membranes with high permeance reduce the required membrane area, while high selectivity improves CO_2_ purity and decreases energy consumption. At a fixed pressure ratio, vacuum pumps offered lower energy use compared to compressors, although they required larger membrane areas, favoring high-permeance membranes for vacuum configurations and high-selectivity membranes for compressor-based systems. The study underscored the need for process-design-informed material development. In a separate study, S. Lee et al. investigated the techno-economic feasibility of low-temperature membrane operation in natural gas combined cycle (NGCC) applications [62]. Their analysis showed that low temperatures improved CO_2_/N_2_ selectivity, enhancing separation performance. Moreover, incorporating Selective Exhaust Gas Recirculation (S-EGR) increased feed CO_2_ concentration and reduced capture cost. A techno-economic analysis (TEA) demonstrated that the cost of CO_2_ capture could be reduced to USD 57 per ton—a 55.1% decrease compared to conventional methods—highlighting the economic viability of low-temperature membrane-based CO_2_ capture systems.

A general comparison of representative CO_2_ capture costs across key technologies is summarized in Table 3. This table outlines typical cost ranges reported in the literature for absorption, adsorption, cryogenic separation, biological capture, and membrane-based systems. It also highlights the main cost-driving factors and typical application domains for each method. While the absolute cost values vary considerably depending on system scale, integration level, and site-specific conditions, the table provides a high-level perspective on the economic positioning of each technology. For example, membrane systems generally offer lower energy requirements and smaller footprints, making them attractive for post-combustion and modular industrial applications. In contrast, cryogenic processes can be cost-effective for high-purity CO_2_ streams but are highly sensitive to energy integration. It should be noted that these cost estimates are indicative and context-dependent, and they do not reflect unified techno-economic benchmarks.

Optimizing membrane-based CO_2_ capture processes requires careful consideration of emission source characteristics to minimize energy consumption. For instance, biogas upgrading typically operates at 3–7 bar, allowing the permeate side to remain at atmospheric pressure and eliminating the need for vacuum pumps. In contrast, syngas from coal gasification is released at much higher temperatures (250–400 °C) and pressures (20–50 bar), necessitating the use of metallic membranes that can withstand such harsh conditions. These variations highlight the need for a systematic understanding of process–performance relationships across different industrial streams. However, despite ongoing advancements in membrane materials, comprehensive reviews focusing on the optimization and demonstration of membrane-based CO_2_ capture tailored to specific emission sources remain limited. In Section 3, we present representative industrial case studies of membrane-based CO_2_ capture, highlighting how system configurations and operating strategies have been tailored to meet the specific conditions of each emission source.

## 3. The Membrane-Based CO_2_ Capture Process in Various Industrial Plants

### 3.1. Pre-Combustion CO_2_ Capture

**Figure 1:**
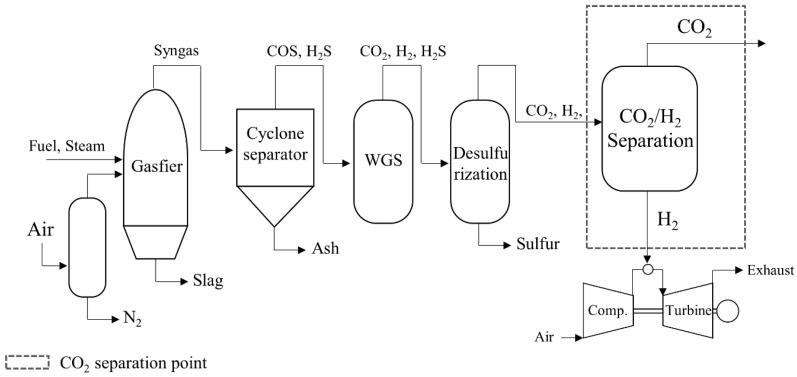
A scheme diagram of IGCC for electricity generation using a gas turbine and the pre-combustion CO_2_ capture method (reproduced from P. Madejski et al. [73], copyright 2022, MDPI).

Pre-combustion CO_2_ capture is employed in processes like coal gasification, Integrated Gasification Combined Cycle (IGCC) power generation, and hydrogen production from fossil fuels like coal and natural gas [73,74]. Examples of these processes are illustrated in Figure 1. In these systems, the fuel is first converted into syngas containing primarily CO and H_2_ through partial oxidation or steam methane reforming. The CO component is then transformed into additional H_2_ and CO_2_ via the water–gas shift reaction. The resulting CO_2_ is subsequently separated, with the primary objective being the purification of hydrogen through selective H_2_/CO_2_ separation. Pre-combustion gas streams typically consist of H_2_ (20–55%), CO_2_ (15–40%), and H_2_O (1–25%), along with trace amounts of N_2_, CH_4_, and H_2_S (each below 1%) [75,76,77,78,79,80]. These mixtures are characterized by high temperatures (250–450 °C) and elevated pressures (20–50 bar), necessitating the use of membrane materials with sufficient thermal and mechanical stability to ensure reliable operation under such demanding conditions.

H. Lin et al., in collaboration with Membrane Technology Research (MTR), conducted a study on H_2_/CO_2_ separation downstream of the water–gas shift (WGS) process in an IGCC plant, using synthesis gas supplied by the University of Kentucky’s Center for Applied Energy Research [81]. The syngas composition included H_2_ (20%), N_2_ (40%), CO (20%), CO_2_ (17%), H_2_O (12%), H_2_S (0.01%), and carbonyl sulfide at 149 ppmv. Prior to membrane separation, a pretreatment stage was applied to remove impurities, such as NO_x_ and SO_x_. The TFC membrane fabricated in the study was palladium (Pd)–polybenzimidazole (PBI)-based MMM. Tests were conducted under feed pressures of 3.4–6.9 bar, a flow rate of 2.4–3 m^3^/h, and a temperature of 40 °C. Initial results showed H_2_ permeance of 146 GPU, CO_2_ permeance of 3.4 GPU, and a selectivity of 42. However, after extended operation, H_2_ permeance decreased to 114 GPU, while CO_2_ permeance increased to 9.3 GPU, reducing H_2_/CO_2_ selectivity to 12. The membrane was designed to achieve 500 GPU of H_2_ permeance and a selectivity of 30. The observed performance degradation was primarily attributed to chemical poisoning from trace contaminants, such as H_2_S and carbonyl sulfide (COS), rather than intrinsic limitations of the MMM structure. This highlights the importance of robust pretreatment strategies and material resistance in pre-combustion CO_2_ capture applications.

Emerson et al. demonstrated stable operation of Pd–Cu alloy tubular membranes for H_2_/CO_2_ separation using syngas generated downstream of the WGS process in a coal gasification system [82]. The gas mixture consisted of H_2_ (50%), CO (1–21%), CO_2_ (12–30%), H_2_O (19–47%), and H_2_S (5–39 ppmv). Under operating conditions of 400–500 °C and 27.6–34.5 bar, the membranes achieved 95% H_2_ recovery and consistent H_2_ production exceeding 0.91 kg/day across four pilot-scale tests. No performance degradation or leakage was observed over 4087 h of continuous operation, even in the presence of H_2_S. However, Pd_4_S formation was identified as a potential degradation mechanism when H_2_S levels exceeded 100 ppmv. Based on these results, the research team proposed scaling up the process to a 45 kg/day hydrogen production system and emphasized the need for further validation under large-scale, long-duration operation.

G. Kirishnan et al. from Stanford Research Institute International evaluated a PBI-based hollow fiber membrane for H_2_/CO_2_ separation using shifted syngas from the WGS process in an IGCC system at the National Carbon Capture Center (NCCC) [83]. The simulated syngas consisted of H_2_ (55%), CO_2_ (41%), CO (1%), H_2_O (1%), and trace components, including N_2_, CH_4_, and 1% H_2_S, following a pretreatment stage for NO_x_ and SO_x_ removal. The simulation was conducted at a feed flow rate of 26.4 Nm^3^/h, feed pressure of 14.8 bar, atmospheric permeate pressure, temperature of 225 °C, and membrane area of 6 m^2^, yielding H_2_ recovery of 95% and CO_2_ purity and recovery of 85% and 90%, respectively. A subsequent field test under similar gas conditions was carried out at 225–250 °C and 41 bar, during which H_2_ permeance was measured at 85–96 GPU, exceeding simulation predictions. The study not only confirmed the feasibility of membrane fabrication and operation but also demonstrated stable performance under industrially relevant conditions.

J. Kniep et al. tested the Proteus^TM^ Gen. 2 multilayer composite membrane for H_2_/CO_2_ separation using shifted syngas from the University of North Dakota’s Energy & Environmental Research facility [84]. The gas mixture contained H_2_ (36.3%), CO_2_ (52.1%), and minor components, including CO, H_2_O, CH_4_, N_2_, O_2_, H_2_S, and hydrocarbons. Bench-scale tests were conducted at feed pressures of 20–35 bar, permeate pressure of 0.7 bar, and temperatures ranging from 130 to 215 °C, with a syngas flow rate of 3.21 Nm^3^/h. At 200 °C and 20.68 bar, the membrane achieved H_2_ permeance of 300 GPU and H_2_/CO_2_ selectivity of 20. A techno-economic analysis (TEA) indicated that the Proteus^TM^ system could reduce the levelized cost of electricity (LCOE) by 7.7% and the CO_2_ capture cost by up to 15% compared to the Selexol process. The study also reported a successful advancement from TRL 4 to TRL 5, highlighting both the performance and scalability of the membrane module. Building on these results, a 50 MW_e_ scale pilot project is planned to further validate the system and improve selectivity for IGCC and hydrogen production applications.

While pre-combustion CO_2_ capture enables efficient separation of H_2_ and CO_2_ under high-pressure, high-temperature syngas conditions, several technological challenges hinder long-term membrane stability. In particular, trace contaminants, such as H_2_S and COS, accelerate membrane degradation, underscoring the need for sulfur-tolerant materials, such as ceramic–carbonate composites or advanced protective coatings [85,86]. In addition, cyclic thermal and mechanical stresses in the 250–450 °C and 20–50 bar range demand deeper understanding of membrane aging mechanisms and the development of thermochemically robust materials for industrial deployment.

### 3.2. Post-Combustion CO_2_ Capture

#### 3.2.1. Natural Gas Power Plant

**Figure 2:**
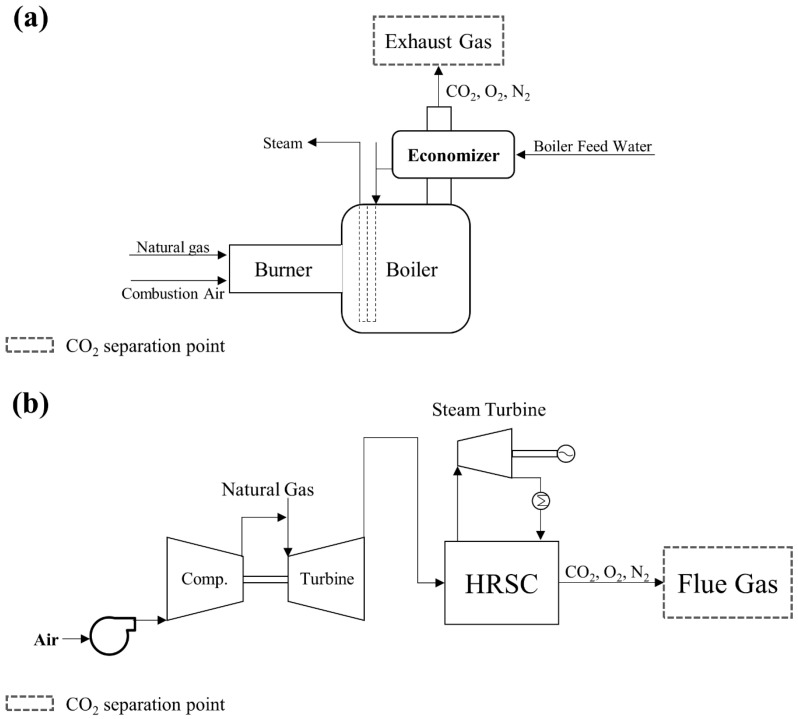
A schematic diagram of (**a**) the natural gas boiler and (**b**) the NGCC process (reproduced from R. W. Baker et al. [89], copyright 2017, Elsevier).

Natural gas power plants have emerged as promising targets for membrane-based CO_2_ capture due to their frequent location in urban areas, where compact system integration is essential, as well as the environmental benefits of solvent-free processes. CO_2_ in these systems is typically captured downstream of heat recovery steam generators (HRSGs) in NGCC units or after boiler exhaust treatment in industrial natural gas (NG) boilers (Figure 2). Although both burn natural gas, the properties of the resulting flue gas differ substantially, which significantly impacts membrane system design and performance.

In NGCC systems, the flue gas passes through a gas turbine, a steam turbine, and HRSG, resulting in low CO_2_ concentrations (3–4%), moderate temperatures (100–150 °C), and near-atmospheric pressure [87]. In contrast, NG boilers emit flue gas directly after combustion and heat recovery, producing higher CO_2_ concentrations (7–10%) and higher temperatures (150–200 °C), which are more favorable for membrane-based separation [69]. Both flue gas components include CO_2_, N_2_, H_2_O, O_2_, NO_x_, and SO_x_. Among these, acidic gases, such as NO_x_ and SO_x_, as well as water vapor can adversely affect membrane performance, and therefore pretreatment steps, such as selective catalytic reduction (SCR), flue gas desulfurization (FGD), and dehumidification, may be required depending on the gas’s composition [88,89]. Therefore, pretreatment steps, such as compression, cooling, and partial impurity removal, should be implemented to ensure compatibility with membrane systems under these conditions. The following case studies illustrate how various membrane technologies have been applied to CO_2_ capture from NGCC and NG boiler flue gases, considering these operational constraints.

W. Ho et al. from Ohio State University demonstrated a membrane-based CO_2_ capture process for NGCC flue gas at the NCCC in Wilsonville, USA [90]. Prior to field testing, the membrane’s performance was evaluated using simulated flue gas composed of CO_2_ (13.0%), N_2_ (55.4%), O_2_ (15.0%), H_2_O (16.6%), and trace levels of SO_2_ and NO_2_ (3 ppm each). Under these conditions, the system achieved a CO_2_ purity of 95.5% and recovery of 91.0%.

Subsequently, a field-scale test was conducted using real NGCC flue gas (CO_2_ 8.6%, N_2_ 86.2%, O_2_ 5.2%, NO_2_ 3 ppm). The feed stream, at a flow rate of 224.4 Nm^3^/h, was compressed to 3.5 bar and cooled to 77 °C before entering a two-stage membrane system. Consistent performance was observed, with 95.5% CO_2_ purity and 91.0% recovery. The system employed multilayer composite membranes incorporating polyvinylamine-based polymers and ionic liquids as CO_2_ carriers, configured in spiral-wound modules. Notably, the ion gel-based membranes maintained high CO_2_ permeance of 4200 GPU even under humid conditions. The membrane surface area was 35 m^2^ in the first stage and 12 m^2^ in the second stage.

J.-H Kim et al. from the Korea Research Institute of Chemical Technology conducted a CO_2_ separation experiment using simulated flue gas to generate design data for a demonstration-scale process targeting 1,000 Nm^3^/h of flue gas from an LNG-fired boiler [91]. The test gas consisted of CO_2_ (12%) and N_2_ (88%), with impurity removal and dehumidification assumed in the pretreatment step. A polyether sulfone (PES) hollow fiber membrane was applied in a four-stage configuration, processing a total of 41.7 Nm^3^/h. Operating pressures were set to 5.88/0.2 bar in the first stage and 2.94/0.98 bar in the second through fourth stages. Under these conditions, the system achieved a CO_2_ purity of 99% while continuously processing 30 Nm^3^/h of flue gas. Simulation results showed good agreement with the experimental data, confirming the validity of the model in predicting separation performance.

Y. Yang and W. Ho et al. from Ohio State University conducted a membrane-based CO_2_ capture test using spiral-wound modules with an effective area of 47 m^2^ at the NCCC [92]. Aminated TFC membranes were fabricated via roll-to-roll casting and coating using a nanoporous PES substrate and a 170 nm thick selective layer composed of poly(N-vinylformamide-co-vinylamine), polyethylene guanidine, and piperazinylethylsarcosinate. The feed gas, derived from a natural gas boiler, consisted of CO_2_ (8.6%), N_2_ (86.2%), O_2_ (5.2%), and 3 ppm of NO_2_. A sulfur dioxide polishing step was included in the pretreatment process. Under permeate pressures of 0.2–0.4 bar and temperatures ranging from 57 to 77 °C, the system achieved CO_2_ purity of 96.5% and recovery of 91.1%. Long-term testing over 1050 h was conducted under varying skid conditions. Throughout this period, the system maintained stable operation with CO_2_ recoveries of 90–99% and purities consistently above 95%. These results confirm the membrane’s durability under extended operation and support its potential for industrial-scale post-combustion CO_2_ capture.

R. W. Baker et al. from MTR conducted a simulation-based study evaluating the use of Selective Exhaust Gas Recirculation (S-EGR) to increase the CO_2_ concentration in NGCC flue gas to 15–20%, thereby enhancing membrane capture feasibility [89]. In the proposed configuration, the retentate stream from the first membrane stage was recycled, with the exhaust directed to a membrane contactor for S-EGR and the permeate stream fed back to the turbine inlet. This approach increased the CO_2_ concentration upstream of the membrane process without requiring major modifications to the existing NGCC system. Economic analysis showed that without S-EGR, the cost of capturing one ton of CO_2_ was USD 60–100 for amine-based systems and USD 55–80 for membrane-based systems. With S-EGR, these costs were reduced to USD 45–65 and USD 44–64, respectively, representing a 20–30% reduction. These findings highlight the potential of S-EGR to improve the economic viability of CO_2_ capture in NGCC applications, addressing one of the key limitations in post-combustion decarbonization.

Due to the low CO_2_ concentration (3–10%) in natural gas flue gas, the single-stage membrane process is limited to achieve both high purity and recovery without incurring excessive energy consumption. To address this limitation, membrane materials must retain high CO_2_ permeability and selectivity under low partial pressure conditions [93]. In parallel, multi-stage configurations incorporating recycle loops should be optimized to reduce energy penalties associated with compressors and vacuum pumps [94]. Furthermore, long-term pilot testing downstream of SCR and FGD units is essential to assess membrane fouling caused by residual NO_x_, SO_x_, and particulates and to develop effective cleaning or replacement strategies [95].

#### 3.2.2. Coal-Fired Power Plant

**Figure 3:**
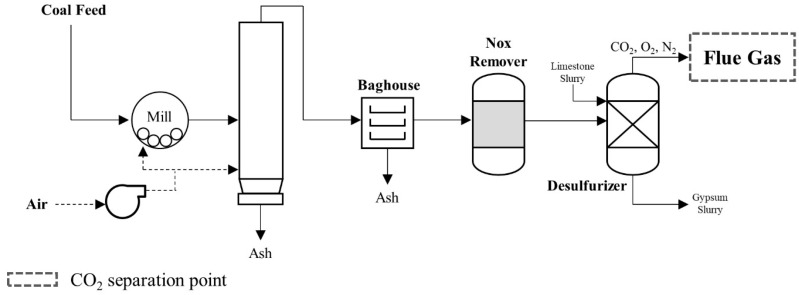
A schematic diagram of a coal-fired power plant process (reproduced from T. A. Adams II et al. [103], copyright 2017, MDPI).

In coal-fired power plants, CO_2_ is produced through the combustion of coal with oxygen and emitted in the flue gas from boilers or kilns (Figure 3). The typical flue gas composition includes CO_2_ (12–14%), N_2_ (67–72%), O_2_ (2–3%), and H_2_O (8–10%), along with various impurities, such as SOx, NOx, CO, and particulate matter [96,97,98]. After combustion, the gas is cooled to 120–180 °C and discharged at atmospheric pressure [99]. The presence of acidic and particulate impurities poses significant challenges for membrane stability, making the chemical resistance of membrane materials a critical requirement [82]. To ensure stable performance, flue gas pretreatment, such as FGD and SCR, is typically implemented upstream of membrane units [100,101,102].

J. Pohlmann et al. from the Karlsruhe Institute of Technology conducted a membrane-based CO_2_ separation test using flue gas from a hard-coal-fired power plant [104]. The flue gas composition included CO_2_ (14.5%), O_2_ (6.5%), H_2_O (14%), SO_2_ (50–100 ppm), NO (76–91 ppm), CO (4–16 ppm), and dust (5–20 mg/m^3^ at STP). Moisture and particulate matter were removed through pretreatment prior to membrane operation. The process employed a single-stage Polyactive^TM^ TFC membrane developed by Air Products operated at 80 °C. Under these conditions, the membrane system achieved CO_2_ purities ranging from 50% to 75% and a recovery rate of 42.7%. Notably, the system maintained stable performance for 740 h despite exposure to acidic and reducing gases. The study further emphasized the importance of robust integration with power plant control systems and the need to ensure system resilience in the event of pretreatment or compression unit failures—key considerations for industrial-scale implementation.

Ebner et al. evaluated a two-stage membrane process for CO_2_ capture from simulated flue gas compositions mimicking those of pulverized coal power plants [105]. The feed gas contained CO_2_ (15%), N_2_ (75%), and O_2_ (10%). The membrane module utilized a thin-film composite configuration with a polysulfone support layer, a polyvinylamine (PVAm) selective layer, and a polydimethylsiloxane (PDMS) protective layer, assembled into spiral-wound modules. Operated at approximately 1.3 bar and room temperature, the system was fed about 12 Nm^3^/h of flue gas. It achieved CO_2_ recovery of ~82% and product purity of ~94%. The design featured retentate recycling from the second to the first stage, enabling enhanced separation performance without significant energy penalty. In terms of economics, the estimated CO_2_ capture cost for this case was around USD 50/ton CO_2_, including compression and operating costs. This suggests that membrane systems may offer a viable alternative to amine scrubbing in post-combustion scenarios under certain integration conditions.

Alex Augustin et al. from Air Liquide Inc. developed a membrane-based CO_2_ capture process using polyimide-based TFC membranes designed for low-temperature operation and cost reduction [106]. The system was tested at the NCCC using flue gas from a coal-fired power plant with a composition of CO_2_ (13.2%), H_2_O (17.3%), N_2_ (66.4%), O_2_ (2.3%), Ar (0.8%), SO_x_ (42 ppmv), and NO_x_ (74 ppmv). The membrane operated under cryogenic conditions ranging from –30 to –45 °C, with feed pressures of 1.2–1.6 bar and permeate pressures of 0.1–0.2 bar. Under cryogenic conditions, 400 Nm^3^/h of flue gas was treated for 500 h, achieving a CO_2_ recovery of 90% and a purity of 58%. A techno-economic analysis estimated the capture cost at USD 32 per ton, confirming the cost-effectiveness and long-term stability of low-temperature membrane systems for coal-fired power plant applications.

T. Merkel et al. from MTR developed and demonstrated a membrane-based CO_2_ capture process for coal-fired flue gas using a 1 ton/day pilot system at the NCCC in Alabama [107]. The system treated flue gas from Plant Gaston Unit 5, which was pretreated by FGD and SCR, and operated at 45–55 °C with a feed flow rate of 250–400 Nm^3^/h for over 1800 h using Polaris^TM^ membrane modules. The process achieved a CO_2_ purity of 40–75% and recovery of over 90% in a two-stage configuration. While high permeance was initially observed, long-term operation revealed gradual performance degradation due to membrane fouling, condensation, and structural damage, indicating the need for periodic maintenance. The study highlighted the importance of optimizing pressure and flow conditions for stable, energy-efficient operation in industrial settings.

In 2024, MTR completed a large-scale membrane-based CO_2_ capture system at the Wyoming Integrated Test Center, designed to treat flue gas from the Dry Fork Station, a coal-fired power plant operated by Basin Electric. The system has a capture capacity of 150 tons of CO_2_ per day and is integrated with a liquefaction unit, producing high-purity liquid CO_2_ (99.9%) at 90% recovery [108]. Building on this deployment, MTR was awarded a Front-End Engineering Design (FEED) contract by the U.S. Department of Energy for a 3 Mt/year capture facility at the same site [109]. Based on these outcomes, MTR has also initiated a membrane-based CO_2_ capture project for a solvent incineration facility operated by the Taiwan Semiconductor Manufacturing Company (TSMC) in Taiwan, with system deployment scheduled by the end of 2025 [110].

While recent studies have demonstrated the feasibility of membrane-based CO_2_ capture in coal-fired power plants, key technical and operational challenges remain. Declines in membrane permeance and selectivity due to residual impurities—despite FGD and SCR pretreatment—highlight the need for corrosion-resistant coatings and protective surface layers. In parallel, the development of highly durable membrane materials capable of maintaining stable performance under high-temperature and high-humidity conditions is essential. Finally, comprehensive techno-economic analyses and process optimization studies are required to enhance energy efficiency and reduce the overall cost of CO_2_ capture at industrial scale.

#### 3.2.3. Cement Industry

In cement production, CO_2_ is primarily generated from the kiln and precalciner stages, driven by the thermal decomposition of limestone (CaCO_3_ → CaO + CO_2_) and the combustion of fossil fuels. The resulting flue gas contains CO_2_ (12–15%), O_2_ (0–21%), N_2_ (balance), H_2_O (0–15%), NO_x_ (~500 mg/Nm^3^), SO_2_ (~50 mg/Nm^3^), CO (~1000 mg/Nm^3^), and dust (10 mg/Nm^3^), and it is discharged at atmospheric or slightly elevated pressure and temperatures of about 150 °C.

Compared to power plant emissions, cement kiln flue gas exhibits greater compositional complexity and variability due to its diverse raw materials and undefined fuel sources. This leads to fluctuating concentrations of acidic gases and particulates, including toxic components, which pose serious risks to membrane performance. As a result, precise and robust pretreatment systems are essential for stable long-term operation. In addition, membrane-based CO_2_ capture technologies must incorporate heat-resistant materials capable of withstanding thermal stress under high-temperature conditions [111,112,113,114].

**Figure 4:**
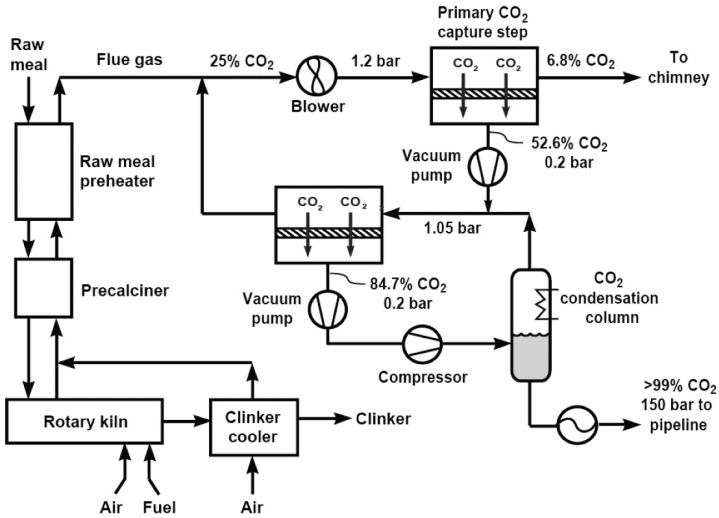
A schematic diagram of a 1000 ton/day cement plant fitted with a membrane CO_2_ capture system (reproduced from R. W. Baker et al. [115], copyright 2018, American Chemical Society).

R. W. Baker et al. from MTR conducted a simulation-based study on membrane CO_2_ capture for cement production, focusing on flue gas emitted from the rotary kiln, the precalciner, and the preheater in a facility generating 1000 tons of CO_2_ per day [115]. The analysis assumed prior removal of NO_x_ and SO_x_ through pretreatment. The simulated gas composition was CO_2_ (20%), O_2_ (4%), and N_2_ (64%). A two-stage membrane configuration with a total membrane area of 2780 m^2^ was applied, operating at 1.2/0.2 bar in the first stage and 1.05/0.2 bar in the second. Across the two stages, the CO_2_ concentration increased from 25% to 84.7%, enabling the recovery of approximately 80% of the plant’s total CO_2_ emissions. A detailed process flow diagram of the capture process is illustrated in Figure 4. This high recovery rate was achieved due to both the elevated CO_2_ concentration in the feed gas, which increased the separation driving force, and the use of high-performance Polaris^TM^ membrane modules developed by MTR. The captured stream was subsequently liquefied to produce high-purity CO_2_, with the overall capture cost estimated at USD 43.2 per ton. These results demonstrate the strong techno-economic potential of membrane-based CO_2_ capture for cement industry applications.

Based on the simulation studies by MTR, T. Merkel et al. developed a preliminary engineering design for a membrane-based CO_2_ capture system [116]. The process was cooperated with Sargent & Lundy LLC (Chicago, IL, USA) and CEMEX Inc. (Houston, TX, USA), targeting flue gas from Kiln 2 at the CEMEX Balcones cement plant. The flue gas’s composition was CO_2_ (14.9%), O_2_ (12.1%), N_2_ (59.0%), and H_2_O (14.0%), with trace SO_2_ (0.37 ppmv), NO_x_ (120 ppmv), and NO_2_ (12 ppmv), emitted at 132 °C and 0.99 bar at a flow rate of 587,791 m^3^/h. A three-stage membrane configuration was proposed, designed to capture 2247 tons of CO_2_ per day—equivalent to approximately 75% of Kiln 2’s emissions. The projected operational lifespan of the system was 25 years, supporting its potential for long-term industrial application. Notably, the assumption of a 25-year operational life highlights confidence in the mechanical and the chemical durability of membrane systems under real flue gas conditions. This design demonstrates long-term, full-scale integration beyond the proof-of-concept stage.

For cement kiln emissions, the same two-stage membrane process developed by Ebner et al. was applied to a simulated flue gas representative of this sector. The feed gas’s composition included CO_2_ (25%), 65% N_2_ (65%), and O_2_ (10%). The tests were conducted at approximately 1.3 bar and ambient temperature, using a feed flow of around 13 Nm^3^/h. The system achieved CO_2_ recovery of around 78% and a final purity of 91%. Due to the lower CO_2_ concentration and the larger membrane area required, the estimated capture cost was higher—approximately USD 53/ton CO_2_. Nevertheless, the results demonstrate that membrane systems remain a technically feasible option for cement flue gas treatment when spatial integration and modularity are prioritized.

These studies demonstrate the practical and economic potential of membrane-based CO_2_ capture in cement manufacturing. However, the slow progress of carbon credit systems, high capture costs, and the risk-averse nature of cement plant operations—particularly as part of critical national infrastructure—remain key barriers to commercialization. Enhancing energy efficiency through strategies like waste heat recovery and process optimization will be essential to improve the viability and accelerate the adoption of membrane technologies in the cement sector.

### 3.3. Iron and Steel Industry

In the steelmaking industry, blast furnace (BF) and basic oxygen furnace (BOF) processes account for approximately 70% of total sectoral CO_2_ emissions [117,118]. The resulting flue gas is characterized by high concentrations of both CO_2_ (17–25%) and CO (20–28%), along with H_2_ (1–5%) and N_2_ (50–55%), distinguishing it from other industrial streams. The CO_2_ capture strategy in steelmaking is generally categorized into (i) direct CO_2_ removal from flue gas, (ii) CO conversion via the WGS reaction followed by capture, and (iii) top gas recycling (TGR), which removes CO_2_ prior to recirculation. Because TGR remains at a relatively early stage of development, it is not discussed in this section. Although chemical absorption using alkanolamine solvents is currently the most commercially adopted CO_2_ capture method in steel plants [118], membrane-based processes remain attractive due to their potential for simpler operation, lower environmental impact, and favorable energy efficiency under moderate-pressure conditions, such as those found in BF gas streams (~3 bar). While membrane-based CCS applications in the iron and steel industry remain limited by lack of demonstration-scale results, this review draws on theoretical studies and simulation-based techno-economic analyses to assess their potential.

Ebner et al. tested flue gas from an integrated steel mill (ISM) to test the versatility of their two-stage membrane capture system. The simulated feed gas consisted of CO_2_ (20%), N_2_ (70%), and O_2_ (10%). The system was operated at room temperature and approximately 1.3 bar, with a feed flow rate of roughly 11 Nm^3^/h. Spiral-wound TFC membrane modules—comprising polysulfone, PVAm, and PDMS layers—demonstrated robust performance. The process achieved approximately 80% CO_2_ recovery with 95% purity. The associated CO_2_ capture cost was estimated at around USD 46/ton CO_2_, benefiting from the moderately higher CO_2_ concentration and the reduced specific energy demand per captured unit. These results underline the applicability of membrane-based capture in steel production environments.

R. W. Baker et al. from MTR conducted a simulation study to evaluate membrane-based CO_2_ capture from steelmaking flue gas, focusing on the BF–BOF route with prior removal of NO_x_ and SO_x_ through pretreatment [115]. The target gas, emitted from blast furnace stoves, had a flow rate of 380,000 Nm^3^/h and a composition of CO_2_ (28.5%), H_2_O (3.8%), O_2_ (2.5%), and N_2_ (65.2%). A two-stage membrane system using Polaris^TM^ modules with a total area of 2780 m^2^ was simulated, operating at feed and permeate pressures of 1.2 and 0.2 bar, respectively, in both stages. The process achieved CO_2_ purity of 71% and recovery of 80%, and the captured stream was further liquefied to >99% purity via condensation at 25 bar. The estimated capture cost was USD 36.0 per ton of CO_2_, demonstrating strong economic viability for membrane deployment in steel manufacturing.

J.-K. Kim et al. from Hanyang University conducted a techno-economic analysis comparing membrane-based and absorption-based CO_2_ capture for industrial flue gas, including the steel industry [119]. Five representative flue gas streams were selected, and process simulations were performed for each case. The results showed that membrane systems become more cost-effective than absorption when the CO_2_ concentration exceeds 30%, while absorption remains favorable at lower concentrations. For example, in a metal production process emitting flue gas with 27.3 mol% CO_2_, the membrane-based capture cost was estimated at USD 41.7 per ton, highlighting its potential under specific process conditions.

The commercialization of membrane-based CO_2_ capture in steelmaking processes requires overcoming several technical challenges, including (i) the development of high-performance membrane materials capable of separating CO_2_ from CO–CO_2_ mixtures [120]; (ii) the optimization of energy consumption in vacuum-driven systems [121]; and (iii) increasing CO_2_ partial pressure at the feed side through integration with the WGS reaction [122]. In addition, membrane durability must be enhanced to tolerate elevated temperatures and acidic impurities, such as SO_x_ and NO_x_, and process scalability must be demonstrated to enable the transition from pilot to full-scale industrial systems.

### 3.4. Biogas Uprading

Biogas is produced through anaerobic digestion (AD) of organic waste streams, such as agricultural residues, landfill material, sewage sludge, and wastewater. The resulting gas typically contains 30–50% CO_2_, which is considerably higher than in flue gas from most industrial processes. In membrane-based upgrading, biogas is compressed to 5–10 bar on the feed side, with the permeate side maintained near atmospheric pressure and operated at ambient temperatures. To withstand these conditions, mechanically robust membranes—commonly made from polysulfone or polyimide—are employed. While the exact composition of biogas varies depending on the feedstock and digestion conditions [123], it generally consists of CH_4_ (55–60%) and CO_2_ (36–40%) as major components, with trace levels of NH_3_, N_2_, O_2_, H_2_, H_2_S, CO, H_2_O, and siloxanes (Table 4) [124]. 

**Figure 5:**
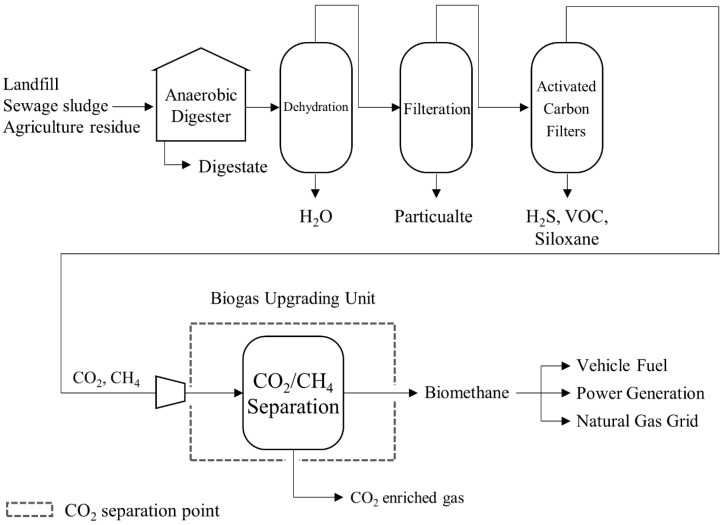
A simplified process diagram of a biogas upgrading plant for biomethane production.

Therefore, an appropriate pretreatment process is required for the impurities’ removal, and the overall process diagram for biomethane production is shown in Figure 5.

Refined biogas can be used for heat, steam, and electricity generation or as a transportation fuel [127]. It can also be compressed into biomethane and injected into existing natural gas distribution networks. Membrane-based biogas upgrading systems have been actively demonstrated and adopted in commercial applications, particularly in Europe, with growing deployment led by companies like Evonik (SEPURAN^®^), Bright Renewables, and Prodeval. These implementations reflect increasing market interest and highlight the need for continued research into performance optimization and broader industrial integration.

V. Vrbová et al. from the University of Chemistry and Technology in Prague demonstrated a membrane-based biogas upgrading system at the Prague Central Wastewater Treatment Plant [123]. The feed gas consisted of CH_4_ (61.8%) and CO_2_ (37.9%), with H_2_S concentrations of 70–100 mg/m^3^ and relative humidity of 40–50%. The membrane modules used in the experiment were asymmetric hollow fiber membranes fabricated from polyimide via condensation polymerization of biphenyl tetracarboxylic dianhydride and aromatic diamines, commercially supplied by UBE Industries. The system operated at 15–25 °C and 6.0–8.0 bar. The process was designed to meet the Czech vehicle fuel standard (≥95% CH_4_), and it successfully achieved CH_4_ product purity exceeding 95%, with CO_2_ purity in the permeate stream reaching 96.4%. The study further emphasized the importance of pretreating H_2_S and water vapor to ensure membrane durability.

P. Wojnarova et al. from the Technical University of Ostrava investigated a spiral-wound polyimide-based TFC membrane system for CH_4_ separation from raw agro-biogas [128]. The membrane, previously shown to tolerate impurities, such as H_2_O, H_2_S, NH_3_, and VOCs, without pretreatment, was designed to swell in the presence of water vapor. Under these conditions, the process achieved CH_4_ purity levels of 95–97%, but CH_4_ recovery was limited to approximately 60% in a single-stage configuration [129,130,131,132]. The study highlighted the need for further research on the relationship between functional layer swelling and operating conditions to enhance the performance of low-cost membrane systems. Notably, membrane swelling was also associated with partial desulfurization of the feed gas, suggesting a dual functionality worth further exploration.

Biomethane produced through biogas upgrading can be used for energy generation—including heat, steam, and electricity—or as a transportation fuel, making it one of the most commercially viable renewable gases to date. Owing to these versatile applications, it has already been deployed in various sectors worldwide. It can also be compressed into bio-compressed natural gas (Bio-CNG) and integrated into existing natural gas distribution networks [127]. Nevertheless, broader implementation is still subject to several technical and economic constraints. These include the high cost associated with impurity removal from sludge-derived biogas, the expense of siloxane removal from landfill gas, and the substantial capital investment required for upgrading systems at wastewater treatment facilities [133,134,135].

## 4. Summary

This review provides a comprehensive overview of membrane-based CO_2_ capture technologies applied across major industrial sectors, including power generation, cement, steelmaking, and biogas upgrading. By consolidating field-tested case studies and process simulations, we analyzed how source-specific characteristics, such as CO_2_ concentration, gas pressure, operating temperature, and impurity composition, directly influence system configuration, membrane applicability, and process performance. For example, CO_2_ concentrations range from 4 to 10% in natural gas power plants to over 30% in biogas and syngas streams, while operating pressures span from atmospheric levels in post-combustion settings to as high as 50 bar in pre-combustion applications (Table 5). These variations necessitate tailored process configurations, such as multi-stage setups, recycle integration, or vacuum-assisted operation, adapted to each emission source.

While early-stage research has predominantly focused on membrane material development, our analysis underscores that actual capture performance depends equally on process-level optimization—including feed composition control, pressure ratio tuning, and pretreatment for impurities, such as SO_x_, NO_x_, and H_2_S. Across various sectors, pilot-scale demonstrations and simulation-based studies have shown that membrane-based CO_2_ capture is technically viable and holds significant potential for commercial application. Demonstrated strengths include low-temperature operation for NGCC flue gas, high-pressure compatibility with syngas and biogas, applicability to CO_2_-rich flue gas in the cement industry, and compact system design suitable for modular or urban-scale deployment.

Membrane-based CO_2_ capture, although promising for its energy efficiency and modular scalability, still faces significant engineering barriers before widespread industrial adoption can be commercialized. A major challenge includes scaling up membrane modules for high-volume flue gas streams, where achieving sufficient recovery and purity often demands multi-stage cascades. These configurations increase both capital expenditure and system complexity. Durability concerns also persist. Polymeric membranes are prone to thermal aging, plasticization, and chemical degradation when exposed to industrial flue gases containing SO_x_, NO_x_, and water vapor. While novel materials, such as ceramic or mixed-matrix membranes, offer improved resistance, their long-term performance under continuous operation remains insufficiently validated.

In addition, membrane systems require large surface areas to process low-CO_2_-concentration gas streams typical of post-combustion applications. This imposes spatial limitations and raises equipment and compression costs, making deployment less feasible in space-constrained environments [136,137].

To address these challenges, current research is focusing on improving membrane performance through simultaneous enhancement of selectivity and permeance, developing chemically robust materials, and standardizing module formats for ease of integration. Demonstration projects at the intermediate scale are also critical to evaluate operational stability and economic viability under realistic conditions [105,136,138,139]. It is our hope that the guidance provided in this review will contribute to overcoming existing technical limitations and advancing practical deployment across diverse emission sectors.

**Table 5:** Separation characteristics of various emission sources [11,12,106].

Emission Source		CO_2_ Conc. (%)	Composition	Pressure (Bar)	Temperature (°C)	References
Pre-combustion	Shifted syngas	15–40	CO_2_, H_2_, H_2_O, N_2_, CH_4_, H_2_S	20–50	250–400	[75,76,77,78,79,80,140]
Post-combustion	Natural gas power plant	4–10	CO_2_, N_2_, H_2_O, O_2_, NO_x_, SO_x_	Ambient	100–200	[88,89,92,141]
	Coal-fired power plant	10–15	CO_2_, N_2_, O_2_, H_2_O, CO, NO_x_, SO_x_, dust	Ambient	90–180	[96,97,98,142,143]
	Cement manufacturing	5–15	CO_2_, N_2_, O_2_, H_2_O, NO_x_, SO_x_, dust	Ambient	100–200	[144,145,146]
Etc.	Steel manufacturing	16–42	CO_2_, N_2_, CO, H_2_	1–3	150–300	[119,147,148]
	Biogas upgrading	30–45	CO_2_, CH_4_, H_2_O, NH_3_, H_2_, O_2_, N_2_, H_2_S, siloxane	3–7	40–60	[124,125,149]

## Figures and Tables

**Table 1:** Industrial stationary CO_2_ emission sources [11,12].

Sources	Processes	CO_2_ Content (%)	Traces
Power plant: pre-combustion	IGCC	30–45	CH_4_, H_2_O, H_2_S, NH_3_
Power plant: post-combustion	Petroleum power plant	6–10	N_2_
	Natural gas power plant	4–8	NO_x_, SO_x_, O_2_
	Coal-fired power plant	10–15	NO_x_, SO_x_, dust
Iron and steel industry	Blast furnace	20–27	CO, H_2_, H_2_S
	Basic oxygen furnace	16–42	
Cement industry	Precalciner	1–30	NO_x_, SO_2_, CO, dust
	Calcination	14–33	
Biogas	Biogas upgrading	30–45	H_2_O, H_2_, O_2_, N_2_, NH_3_, H_2_S, siloxane

**Table 2:** Comparison of carbon dioxide capture technologies [1,14,15,16,17,18,19,20,24,25].

Method	Concept	Advantage	Disadvantage
Absorption	Dissolves CO_2_ using chemical solvent (e.g., amines)	Effective for large-scale emission sources	Solvent corrosion and regeneration costs
Adsorption	Captures CO_2_ using porous material (e.g., zeolites, activated carbon)	Regenerable adsorbents	Requires high thermal energy
Cryogenic Separation	Liquefies CO_2_ at cryogenic temperature for separation	Produces high-purity CO_2_	High energy consumption
Biological Capture	Uses microorganisms, algae, or enzymes to absorb CO_2_	Environmentally friendly	Slow absorption rate, requires large space
Membrane	Separates CO_2_ using selective permeation through membranes	Flexible operation, low energy consumption, easy to modularize	Impurity sensitivity, limited performance at low CO_2_ concentrations

**Table 3:** Representative CO_2_ capture costs and application contexts by technology.

Method	Cost Range (USD/Ton CO_2_)	Key Factors Affecting Cost	Primary Applications	References
Absorption	USD 44–71	Solvent properties, energy requirements, plant scale	Power plants, industrial flue gas treatment	[63,64]
Adsorption	USD 37–57	Adsorbent characteristics, regeneration method, energy penalty	Industrial point sources, moderate CO_2_ concentrations	[65,66]
Cryogenic Separation	USD 12–150	Process integration, energy efficiency, scale	High-purity CO_2_ streams, industrial gas processing	[67,68,69]
Biological Capture	USD 88–116	Biomass availability, co-product value, scale	Power generation with negative emissions	[63,70]
Membrane	USD 42–50	Compression and vacuum energy, membrane fabrication, module costs	Post-combustion capture, industrial flue gas	[71,72]

**Table 4:** Composition of various biogas sources [125,126].

Component	Agricultural Residue	Sewage Sludge	Landfill	Wastewater
CH_4_ (%)	50–70	60–70	35–65	55–58
CO_2_ (%)	25–45	34–38	30–45	32–50
H_2_O (%)	1–6	1–7	1–5	1–5
H_2_ (%)	Traces	Traces	0–5	Traces
O_2_ (%)	0–1	Traces	0–1	Traces
N_2_ (%)	0–5	0–2	5–15	Traces
NH_3_ (ppm)	0–100	50–100	0–5	0–100
H_2_S (ppm)	0–1000	0–400	0–100	0–4000
Siloxane (%)	0–0.2	0–0.2	0–0.2	0–0.5

## Data Availability

No new data were created or analyzed in this study.

## Abbreviations

The following abbreviations are used in this manuscript:

AD	Anaerobic Digestion
BF	Blast Furnace
Bio-CNG	Bio-Compressed Natural Gas
BOF	Basic Oxygen Furnace
COS	Carbonyl Sulfide
FEED	Front-End Engineering Design
FGD	Flue Gas Desulfurization
FTM	Facilitated Transport Membrane
HRSG	Heat Recovery Steam Generator
IGCC	Integrated Gasification Combined Cycle
LCOE	Levelized Cost of Electricity
MMM	Mixed-Matrix Membrane
MTR	Membrane Technology Research
NCCC	National Carbon Capture Center
NG	Natural Gas
NGCC	Natural Gas Combined Cycle
PBI	Polybenzimidazole
PDMS	Polydimethylsiloxane
PES	Polyether Sulfone
PVAm	Polyvinylamine
SCR	Selective Catalytic Reduction
S-EGR	Selective Exhaust Gas Recirculation
TEA	Techno-Economic Analysis
TFC	Thin-Film Composite
TGR	Top Gas Recycling
WGS	Water–Gas Shift
